# Effect of *In Vitro* and *In Vivo* Anakinra on Cytokines Production in Schnitzler Syndrome

**DOI:** 10.1371/journal.pone.0059327

**Published:** 2013-03-19

**Authors:** David Launay, Virginie Dutoit-Lefevre, Emmanuel Faure, Olivier Robineau, Carine Hauspie, Vincent Sobanski, Eric Hachulla, Myriam Labalette, Pierre-Yves Hatron, Sylvain Dubucquoi

**Affiliations:** 1 Lille Nord de France University, Department of Internal Medicine and Clinical Immunology, National Reference Center for Rare Autoimmune and Systemic Diseases; Regional University Hospital, Lille, France; 2 EA2686, IMPRT IFR 114, Regional University Hospital, Lille, France; 3 Lille Nord de France University, Department of Immunology, Regional University Hospital, Lille, France; 4 Lille Nord de France University, Pseudomonas Aeruginosa Host-Pathogen Translational Research Group, Regional University Hospital, Lille, France; University of California, Riverside, United States of America

## Abstract

IL-1 receptor antagonist anakinra is usually highly efficient in Schnitzler syndrome (SS), a rare inflammatory condition associating urticaria, fever, and IgM monoclonal gammopathy. In this study, we aimed to assess lipopolysaccharide (LPS)-induced production of inflammatory cytokines by peripheral blood mononuclear cells (PBMCs) before and after 1 month of anakinra in patients with SS. LPS-induced production of IL-1β, IL-6 and TNFα was assessed by enzyme-linked immunosorbent assay with and without anakinra *in vitro,* and before and after 1 month (*in vivo* condition) of treatment in 2 patients with SS. Spontaneous production of IL-1β, IL-6 and TNF-α by PBMCs was similar in the patients and the healthy controls and was almost undetectable. Stimulation with LPS caused a higher release of cytokines from the patients than from the healthy controls. Before *in vivo* anakinra start, *in vitro* adjunction of anakinra reduced the high LPS-induced production of IL-1β and TNFα in both patients and of IL-6 in one patient. After 1 month of treatment with anakinra, while the patients had dramatically improved, there was also a marked reduction in LPS-induced cytokines production, which was almost normalized in one patient. This study shows an abnormal LPS-induced inflammatory cytokines production in SS, which can be decreased or even normalized by *in vitro* and *in vivo* anakinra.

## Introduction

Schnitzler syndrome (SS) is a rare inflammatory condition characterized by urticarial-like rash, fever and monoclonal IgM gammopathy [1]. A role of interleukin (IL)-1β has been suggested [2], [3], [4] and IL-1 receptor antagonist anakinra was tried successfully in SS on the basis of its efficacy in treating some hereditary autoinflammatory syndromes, especially cryopyrin-associated periodic syndrome [5], [6], [7]. This supported the hypothesis that the inflammasome could play a crucial role in these diseases [8], [9], [10], [11], [12], [13]. Studies on cytokines production in SS patients are scarce and *in vitro* and *in vivo* effects of anakinra on their production are not known [14], [15], [16]. We report 2 cases of SS successfully treated with anakinra. We determined, before and during treatment with anakinra, IL-1β, IL-6 and TNFα plasma levels and *in vitro* production by PBMCs of these cytokines with and without stimulation by lipopolysaccharide (LPS) and with and without *in vitro* anakinra.

## Methods

### Cytokines Concentrations Assays

Heparinized blood samples were obtained from two SS patients before and one month after starting subcutaneous 100 mg/day anakinra (*in vivo* condition) and from six healthy subjects (HSs) between January 2009 and January 2011. The blood sample obtained after one month of treatment with anakinra was taken 24 hours after the last anakinra injection and just before the next one. SS patients and healthy subjects gave written informed consent to take part in the study, which was approved by our IRB (Comité de Protection des Personnes, Nord). PBMCs were isolated by density centrifugation on Ficoll-Paque PLUS (GE Healthcare, Uppsala, Sweden). Cells were maintained in 24-well flat-bottomed plates with a final density of 1×10^6^/ml in a volume of 1 ml RPMI 1640 including 10% fetal bovine serum or patient plasma, 1% penicillin/streptomycin (Life Technologies, Cergy Pontoise, France) PBMCs were incubated with or without 100 pg/ml LPS (Sigma-Aldrich, Lyon, France) and with (“*in vitro*” anakinra condition) or without 500 ng/ml anakinra (SOBI). After 6 and 16 hours, supernatants were collected and stored at −80°C. Concentrations of IL-1β, IL-6 and TNF-α in plasma and supernatant were measured by enzyme-linked immunosorbent assay (Life Technologies, Cergy Pontoise, France). Results are expressed as the mean of triplicate measures of cytokine concentration (± standard deviation).

### RNA Quantitative Real-time qPCR

PBMCs of 3 healthy subjects and of the second patient with SS, obtained before and 1 month after the start of anakinra, were kept at 80°C in microcentrifuge tubes. Extractions of RNA in these unstimulated PBMCs were performed using GeneJet RNA Purification Kit (Fermentas, K0731). Analyses were performed using AbiPrism 7500 Lighcycler system using 0.25 µM concentrations of specific primers and SYBR Green II PCR Master Mix (Qiagen). Specific primers for: HBMS forward (5′-GGCAATGCGGCTGCAA-3′) and reverse (3′-GGGTACCCACGCGAATCAC-5′); IL-1ß forward (5′-AAA-CCTCTTCGAGGCACA-AG-3′) and Reverse (3′-GTTTAGGGCCATCAGCTTCA-5′); IL-6 forward (5′-GGTACATCCTCGACGGCATCT-3′) and Reverse (3′- GTGCCTCTTTGCTGCTTTCAC-5′) were used for amplification in duplicate assays. PCR amplification was performed to control for sample loading and to allow normalization between samples. Quantitative comparison was obtained through the comparative Ct method.

## Results

### Description of Patient 1

A 54-year-old male was referred to our institution with a 5-year history of recurrent episodes of urticarial lesions associated with fever up to 39°C. He also complained of relapsing arthralgia and bone pain. At his admission, laboratory investigations showed neutrophilic leukocytosis at 12×10^9^/L, and increased C-reactive protein (CRP) (6.0 mg/dl, N<0.3). IgM-κ monoclonal gammopathy was also found. Tests for autoimmunity, infections and malignancy were negative. Based on these findings, we diagnosed SS [^1^]. Anakinra was started at a daily dose of 100 mg. Within 48 hours, the patient had a dramatic clinical/biological response, with the resolution of skin symptoms and bone pain and normalization of CRP. During the following year, the patient experienced no episode of urticarial lesions and CRP was normal under treatment.

### Description of Patient 2

A 77-year-old female was referred to our institution with a similar history than patient 1, i.e. a 6-year history of recurrent episodes of urticarial lesions associated with fever up to 40°C. Her medical history included a severe osteoporosis. She also experienced relapsing bone pain. Laboratory investigations showed neutrophilic leukocytosis at 17×10^9^/L, and increased CRP (14.0 mg/dl, N<0.3). IgM-κ monoclonal gammopathy was also found. Tests for autoimmunity, infections and malignancy were negative. Based on these findings, we diagnosed SS [^1^]. Anakinra was started at a daily dose of 100 mg. Within 48 hours, the patient had a dramatic clinical/biological response, with the resolution of skin symptoms and bone pain and normalization of CRP. During the following year, the patient experienced no episode of urticarial lesions and CRP was normal under treatment.

### Production of Cytokines before Treatment, with and without *in vit*ro anakinra

**Figure 1:**
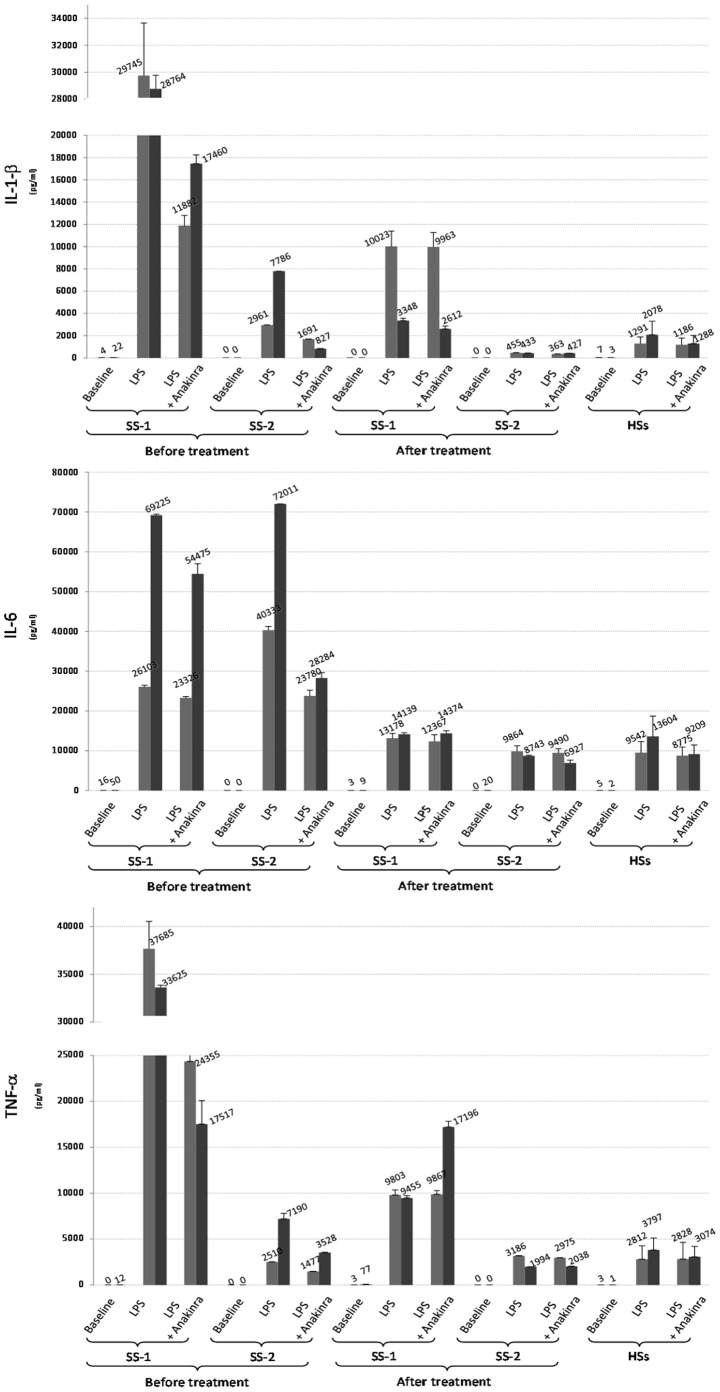
Production of IL-1β, TNF-α and IL-6 by PBMCs of 2 Schnitzler syndrome (SS-1 for patient 1 and SS-2 for patient2) patients and 6 healthy subjects (HSs) in different conditions of culture. (i) baseline: no stimulation condition of PBMCs; (ii) LPS stimulation (100 pg/ml); (iii) LPS stimulation (100 pg/ml) plus 500 ng/ml anakinra *in vitro.* Release of cytokine was evaluated in the supernatants collected at both 6 hours (middle gray histograms) and 16 hours (dark gray histograms) of culture conditions. Results are expressed as the mean of triplicate measures of cytokine concentration (± SD). In the SS patients, cytokine release was measured before and 1 month after *in vivo* anakinra treatment. In HSs, cytokine release is expressed as the mean of values (+/− SD) obtained in 6 independent evaluations.

Spontaneous production of IL-1β, IL-6 and TNF-α by PBMCs was similar in the patients and the healthy controls and was almost undetectable (Figure 1 baseline level), as were plasma levels of cytokines (data not shown). Stimulation of the patients PBMCs with their own plasma induced a negligible secretion of cytokines (data not shown). Before the first administration of anakinra, 6 and 16 hours of stimulation with LPS caused a larger release of cytokines by PBMCs from the patients than from the healthy controls (Figure 1) but with different patterns between the patients. In patient 1, all 3 cytokines were released at a very high level when compared to healthy controls. In patient 2, although IL-1β and TNF-α levels were higher than in healthy controls, they were lower than in patient 1 and the peak of production was delayed (6 hours for patient 1 and at least 16 hours for patient 2). Conversely, IL-6 production was quite similar between both patients and much higher than in healthy controls. Adding anakinra *in vitro* reduced LPS-induced secretion of IL-1β and TNF-α after 6 and 16 hours in both patients but had no obvious effect on IL-6 in patient 1 while it decreased for more than 50% IL-6 production in patient 2 (Figure 1).

### Production of Cytokines after 1 Month of anakinra Treatment with or without *in vitro* anakinra

One month after the initiation of *in vivo* anakinra treatment, the release of cytokines was dramatically decreased both after 6 and 16 hours of stimulation with LPS in both patients and almost normalized in patient 2. The most impressive decreases were observed for LPS-induced IL-1β production after 16 hours of stimulation in patient 1 which was close to healthy controls, and for LPS-induced IL-6 production after 16 hours of stimulation in patient 2 which was also close to healthy controls. LPS-induced IL-1β release observed after 6 hours of stimulation for patient 1 and 16 hours for patient 2 was similar to the release, which was observed after 6 hours with *in vitro* anakinra before treatment in patient 1 and 16 hours in patient 2. When the patients PBMCs obtained after 1 month of anakinra treatment were incubated with additional anakinra *in vitro*, we did not observe any additional effect on cytokine production (Figure 1).

### Gene Expression of IL-1β and IL-6 before and after 1 Month of anakinra (Figure 2)

Before the start of anakinra, spontaneous gene expression of IL-1β, but not of IL-6, in unstimulated PBMCs was increased in the patient with SS when compared to healthy subjects. After 1 month of treatment with anakinra, gene expression of IL-1β in unstimulated PBMCs of the patient with SS was not different from the healthy subjects. Gene expression of IL-6 remained unchanged and similar to the healthy subjects.

**Figure 2:**
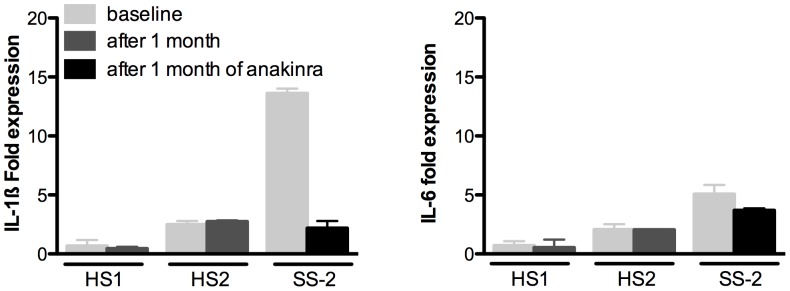
Gene expression of IL-1β and IL-6 in unstimulated PBMCs by RNA quantitative real-time qPCR in the second patient with Schnitzler syndrome (SS-2) and healthy subjects (HS1 and HS2), at baseline (i.e. before the start of anakinra) and after 1 month of treatment with anakinra.

## Discussion

In accordance with previous studies, we showed that plasma levels as well as spontaneous production by PBMCs of IL-1β, IL-6 and TNF-α were similar in the SS patients and the healthy controls [14], [15], [16], [17]. These results contrast with the frequent elevated spontaneous IL-1β production in some other autoinflammatory syndromes like the cryopyrin-associated periodic syndrome [5], [6], [7] and suggests that plasma level determination of these cytokines are not useful in the diagnosis and the follow-up of SS patients. Most studies showed that LPS alone was sufficient to overactivate IL-1β secretion in SS, suggesting an abnormal activation of the intracellular processes leading to production and secretion of inflammatory cytokines, perhaps through inflammasome-dependent mechanisms [15], [16], [17]. Conversely, one study suggested that in some SS patients LPS could not increase the inflammatory cytokines production [18]. Our study adds some line of evidence that LPS does induce an abnormal release of proinflammatory cytokines IL-1β, IL-6 and TNF-α by PBMCs in SS, with different kinetic patterns and magnitude according to the patients [14], [15], [16], [17]. In patient 1, all the 3 cytokines were produced at a high level after 6 hours of LPS stimulation. In patient 2, cytokines production after 16 hours of LPS was higher than after 6 hours and was very high for IL-6. These interindividual variations in cytokine responses observed in our patients had to be expected and may be explained by polymorphisms as well as gender and age differences [19]. The high production of IL-6 after LPS stimulation is of particular interest since a recent study reported a complete remission after anti-IL-6 treatment in 3 patients who failed anti-IL-1β treatment [18]. We observed that stimulation of the patients PBMCs with their own plasmas induced a negligible secretion of cytokines. This argues against the existence of a stimulatory factor in the plasma in SS [3].

Although the spontaneous production of IL-1β and IL-6 by unstimulated PBMCs of SS patients was very low and not different from healthy subjects, we show that spontaneous IL-1β but not IL-6 gene expression was markedly increased in unstimulated PBMCs of SS patient when compared to healthy subjects. This spontaneous high gene expression of IL-1β in unstimulated PBMCs in SS could explain why LPS alone can trigger an efficient release of cytokine. Indeed, these findings suggest that PBMCs in SS patients have already undergone a first priming from extrinsic or intrinsic origin that allows substantial IL-1 β release in the absence of the ‘second hit’ usually required to trigger efficient release [15]. The fact that the spontaneous gene expression of IL-6 in PBMCs was similar in SS patient and healthy subjects while IL-6 production after LPS stimulation was much higher than in healthy controls suggests that IL-6 production could be dependent on IL-1 β stimulation, by an amplification loop process.

We report here for the first time the effects on LPS-induced cytokines release of anakinra both *in vitro* and after 1 month of subcutaneous *in vivo* administration in two SS patients. The direct and rapid *in vitro* effect of anakinra on IL-1β suggests that there is an inhibition of the known autostimulatory loop of IL-1β production [20], [21]. In keeping with this hypothesis, we show that the spontaneous gene expression of IL-1β in PBMC after 1 month of anakinra was normalized in SS patient 2 [21]. IL-1 trap (an inhibitor of IL-1) has also been shown to reduce IL-1β, IL-6 and TNF-α production by LPS-stimulated PBMCs in SS [14]. These *in vitro* results suggest that inhibiting the IL-1 autostimulatory loop [21] by blocking IL-1 receptors, can also inhibit TNF-α production in SS. Patterns of response differed between the 2 patients for IL-6 as its production was markedly decreased after *in vitro* anakinra in patient 2 and less impressively in patient 1.

After 1 month of anakinra treatment, we observed a dramatic decrease in IL-1β production. For patient 1, after 16 hours of LPS stimulation, IL-1β release was ten-fold lower than IL-1β release observed at baseline and within range of the values observed in the healthy controls, as was IL-6 production. In patient 2, IL-1β, IL-6 and TNF-α production after LPS stimulation was similar to the healthy controls, suggesting that anakinra had restored a normal phenotype of LPS response in SS patients. Interestingly, dramatic decrease of IL-6 production after 1 month of anakinra treatment was confirmed by normal CRP values in both patients. In cryopirin associated periodic syndromes, *in vivo* anakinra also decreased the production of IL-1β by LPS-stimulated PBMCs [8], [12], [22], but *in vitro* data are lacking. The release of IL-6 and TNF-α was lower than with anakinra *in vitro* before treatment. This suggests that IL-6 and TNF-α production is also dependent on the long-term action of IL-1β on PBMCs rather than the immediate effect of IL1 receptor blockade. We also have to discuss here why we chose to obtain the blood sample 24 hours after the last anakinra injection and not just after. The aim of our study was to assess the long term and not the acute effect of *in vivo* anakinra on cytokines production in SS. By showing that even 24 hours after the last injection, LPS-stimulated cytokines production was improved in our patients (and even almost normalized in one SS patient), we suggest that anakinra is able to restore an almost normal PBMC phenotype in SS. Altogether, these results could explain the dramatic improvement of the clinical manifestations after the start of anakinra. When the patients PBMCs obtained after 1 month of anakinra *in vivo* were incubated with additional anakinra *in vitro*, we did not observe any additional effect on cytokine production Whether this result could translate in indications of uptitrating or not the dose of anakinra needs further study.

Our study has some limitations. It involves a limited number of patients. However, SS is a very rare condition and studies focusing on cytokine production have included between 1 and 3 patients. To better understand the mechanisms of abnormal LPS-induced cytokine productions, it would also have been interesting to examine the levels of IL-1β in cells lysates to determine whether elevated pro-IL-1 levels were readily present in patient’s PBMCs leading to higher cytokine release upon inflammasome-dependant activation. The use of ultrapure LPS and/or other inflammasome- activators could also allow them to better understand the mechanisms involved in the enhanced release of IL-1β by patient’s PBMCs. IL-18 and IL-1α would also have been interesting to focus on.

Our study confirms the pivotal role of IL-1β in SS and shows that the marked efficacy of anakinra on clinical symptoms is associated with a dramatic decrease of abnormal IL-1β, IL-6 and TNF-α production by LPS-stimulated PBMCs. Whether *in vitro* and *in vivo* effect of a treatment on cytokine production in SS could help anticipating its efficacy or failure warrants further studies.
